# How E_2_ Induces Uterine Effects: Transcription Coordinates Cascade

**Published:** 2004-11

**Authors:** Julia R. Barrett

The rodent uterotrophic assay, a standard method for assessing a compound’s estrogenicity, offers a model for phenotypic anchoring, or linking changes in gene expression to specific pathologic changes. Typically, when an immature rodent uterus is exposed to the endogenous estrogen 17β-estradiol (E_2_), it undergoes cell proliferation and differentiation that can be measured through weighing and histological analysis. The uterine changes triggered by estrogens are directed by numerous genes, but little has been known about the molecular events involved and how they relate to observable physical change. A wealth of detail is now provided through research by Jonathan Moggs of Syngenta’s Central Toxicology Laboratory in the United Kingdom and colleagues **[*EHP* 112:1589–1606]**. According to the team’s findings, E_2_ induces a highly coordinated transcriptional program that orchestrates a cascade of cellular events related to uterine growth.

The scientists’ findings are based on a standard rodent uterotrophic assay. Female mice were given a single E_2_ or control injection at approximately 3 weeks of age and then euthanized at specified time intervals (1, 2, 4, 8, 24, 48, or 72 hours). After the animals’ uteri were weighed, samples were taken for histological analysis, and remaining tissue was subjected to RNA extraction for microarray analysis.

The researchers confirmed the physical events of this typical assay. Uterine weights began to change rapidly after the E_2_ injections. A significant increase was seen by 4 hours, with maximum weight gain reached at 24–72 hours. Cellular changes were also rapid. By 4 hours after injection, the stromal endometrium had thickened due to water uptake; cell growth and proliferation were apparent between 8 and 24 hours.

Total RNA was isolated from the pooled uteri for each treatment group, and labeled complementary RNAs were constructed and hybridized to microarrays to yield 42 data sets. Analysis of gene expression led to the identification of 3,538 E_2_-responsive genes. Further analysis allowed the grouping of these genes into coregulated clusters and the identification of the predominant gene functions associated with each cluster. Finally, by comparing gene expression and changes in uterine weight and histology with regard to time, the scientists were able to anchor changes in gene expression to changes in uterine characteristics.

These new microarray data reveal that the interaction of an exogenous estrogen with estrogen receptors initiates a highly coordinated molecular cascade that drives uterine growth and cell differentiation. The molecular program begins with the induction of genes that regulate transcription and signal transduction. It continues with the regulation of genes involved in protein biosynthesis, cell proliferation, and epithelial cell differentiation. Other gene functions are interwoven into the program, including the direction of fluid uptake and coordination of cell division.

With regard to time, changes in gene expression and uterine characteristics fell into four distinct phases. In the first phase, covering the first 4 hours after injection, E_2_ rapidly induced transcriptional regulators and signaling components for a multitude of pathways, including those responsible for regulating fluid influx. The second phase, 4–8 hours after injection, was characterized by induction of genes needed for mRNA and protein synthesis, but no changes in physical uterine characteristics. During the third phase, occurring 8–24 hours after injection, uterine weight doubled, and cells entered the replication cycle, while genes controlling chromosome regulation and cell cycle were under active regulation. Finally, in the fourth phase, 24–72 hours following E_2_ exposure, the genes being induced were those involved in uterine cell differentiation and defense responses.

The researchers write that their findings provide a basis for understanding the mechanisms by which other estrogenic compounds, including environmental chemicals, induce their effects. Also, the large number of E_2_-responsive genes that they identified provides an array of potential marker genes that could be useful in short-term estrogenicity assays. Finally, the scientists note that their work provides a paradigm for understanding the mechanisms of action for estrogen as well as other nuclear receptors.

